# Self-regulation in the pre-adolescent brain

**DOI:** 10.1016/j.dcn.2021.101012

**Published:** 2021-09-10

**Authors:** P. Pas, H.E. Hulshoff Pol, M. Raemaekers, M. Vink

**Affiliations:** a UMCU Brain Center, University Medical Center Utrecht, University Utrecht, Utrecht, The Netherlands; b Developmental Psychology, Utrecht University, Utrecht, The Netherlands; c Experimental Psychology, Utrecht University, Utrecht, The Netherlands

**Keywords:** Self-regulation, Inhibition, Children, Development, fMRI, **YOUth Cohort Study**

## Abstract

Self-regulation refers to the ability to monitor and modulate emotions, behavior, and cognition, which in turn allows us to achieve goals and adapt to ever changing circumstances. This trait develops from early infancy well into adulthood, and features both low-level executive functions such as reactive inhibition, as well as higher level executive functions such as proactive inhibition. Development of self-regulation is linked to brain maturation in adolescence and adulthood. However, how self-regulation in daily life relates to brain functioning in pre-adolescent children is not known. To this aim, we have analyzed data from 640 children aged 8–11, who performed a stop-signal anticipation task combined with functional magnetic resonance imaging, in addition to questionnaire data on self-regulation. We find that pre-adolescent boys and girls who display higher levels of self-regulation, are better able to employ proactive inhibitory control strategies, exhibit stronger frontal activation and more functional coupling between cortical and subcortical areas of the brain. Furthermore, we demonstrate that pre-adolescent children show significant activation in areas of the brain that were previously only associated with reactive and proactive inhibition in adults and adolescents. Thus, already in pre-adolescent children, frontal-striatal brain areas are active during self-regulatory behavior.

## Highlights

• Children with higher levels of self-regulation employ more proactive inhibition.
• During proactive inhibition, children aged 8–11 show activation in frontal-cortical areas.
• Children higher in self-regulation exhibit more cortical-subcortical coupling.
• Children aged 8–11 show similar brain activation as adults during inhibition.

## 1 Introduction

To function adequately in everyday life, the ability to effectively exert control over your emotions, behavior, and impulses is crucial. This capacity is commonly referred to as self-regulation. Self-regulation has been defined as the ability to monitor and modulate emotions, behavior, and cognition, that in turn allows us to achieve goals and adapt to changing circumstances (Berger et al., 2007). This capacity develops from early infancy until well into adulthood. Where goals early in life are concrete and focused on direct rewards, e.g. food and nurture, there is a shift in adolescence where the ability arises to forgo immediate gratification and goals gradually become more abstract and long-term (Mischel et al., 1989).

Self-regulation can be studied across development in terms of executive functions (Vink et al., 2020). One such executive function is inhibition, the ability to suppress behavioral responses, and develops in infancy and preschool years (Diamond*,* 2013). During middle childhood, children develop high-level executive functions, such as planning, problem solving, information processing and cognitive flexibility (Rosario Rueda et al., 2019). These high-level executive functions are founded on the integration of low-level functions. Then, during adolescence, the various executive functions start becoming integrated to support high-level executive control, also called cognitive control (Anderson et al., 2001). Executive control refers to the coordination of previously acquired low- and high-level executive functions such as working memory, inhibition, mental shifting, and information processing, which are then called upon as needed (Friedman et al., 2008, Best et al., 2011).

In the case of inhibition, it has been shown that while children at the end of childhood can inhibit prepotent responses, a low-level executive function, they further develop this skill during adolescence (Vink et al., 2014). This improvement is associated with the rise of proactive response strategies that allow for more efficient processing by engaging inhibitory functions prior to the actual inhibition, leading to the anticipatory slowing down of responses (Zandbelt and Vink, 2010, Pas et al., 2017, Pas et al., 2019). The true progress across childhood and adolescence is not better executive functions in itself, but rather more effective use of these functions due to their integration with other high-level executive functions such as planning. As such, the development of self-regulation is supported by the development of low-level executive functions early in life, and their subsequent integration later on (Vink et al., 2020).

This integration of executive functions, which allows for proactive inhibitory control, has been theorized to depend upon the establishment of frontal control over the rest of the brain, in particular subcortical regions (Cools, 2011, Vink et al., 2014, Insel et al., 2017). The shift from low-level reactive to more higher-level proactive inhibition strategies has previously been linked to increased frontal activation as well as increased functional coupling between frontal and subcortical regions (Vink et al., 2014, Van den Bos et al., 2015).

However, these previous studies included adolescents and young adults, and we therefore do not yet know if and how individual differences in the state of executive function development and brain maturation pre-adolescence are linked to levels of self-regulation. It may very well be that children who show higher levels of self-regulation are better able to engage relevant brain regions during the execution of executive functions. This may be coupled with increased functional coupling between regions that will begin to form brain networks. For instance, stronger frontostriatal connections have been linked to better delay of gratification (Achterberg et al., 2016). Consequently, measurements at this period in development may provide predictors of progress in adolescence and possibly outcome in adulthood. There have been some studies linking inhibitory control in children to brain measures, but their sample sizes are either relatively small (Durston et al., 2002, Schel et al., 2014, Steinbeis et al., 2015, Liuzzi et al., 2020), focused on inhibition and unhealthy eating (English et al., 2019, Van Meer et al., 2016), or used samples of at-risk children (Ware et al., 2015, Réveillon et al., 2016, Van Hulst et al., 2018, Meldrum et al., 2018, Cope et al., 2020). Our aim is to investigate whether there are associations between self-regulation and brain measures in a large cohort of typically developing children. We will assess children's self-regulatory abilities in daily life via questionnaires. These data will be combined with self-regulatory measures from an inhibition task and accompanying functional MRI measures, that include both low-level response inhibition and higher-level anticipatory processes. This allows us to investigate to what degree individual differences in the brain areas underlying inhibitory control exist, whether they are linked to self-regulation in daily life, if this changes with age and whether this is different for boys and girls. Adolescent males are known to exhibit higher levels of sensation seeking and lower levels of impulse control than females, and those differences even appear during pre-adolescence (Shulman et al., 2014).

The central hypothesis is that children who show high levels of self-regulation in daily life will also show higher levels of reactive and proactive inhibitory control. Behaviorally, we expect children scoring higher on self-regulation, to demonstrate more proactive inhibitory control during the task, resulting in the slowing down of responses in anticipation of a stop-signal on go trials (Pas et al., 2019, Vink et al., 2014). In the brain, we expect this measure to be associated with the establishment of frontal control over the rest of the brain (Cools, 2011). This is expected to result in higher levels of activation in the right mid frontal cortex (Pas et al., 2019), and increased functional coupling between cortical and subcortical regions. Specifically, between the right frontal cortex and the striatum during proactive inhibition, and the left-motor cortex during reactive inhibition (Vink et al., 2014). To investigate these specific hypotheses, we will be using a region-of-interest based approach employing the corresponding regions from previous research.

## 2 Materials and methods

### 2.1 Participants

We requested the largest available data sample from the YOUth cohort study (Onland-Moret et al., 2020), which provided us with a total of 798 subjects. There is currently no data available from children performing our current fMRI inhibition task that allows us to conduct a power analysis. However, we opted for this considerable number because functional MRI in children can lead to moderately reliable results, due to suboptimal task-compliance and movement (see Buimer et al., 2020), and a higher number allows us to investigate subtle differences in brain activation. Of all subjects a complete anatomical and functional scan was available, as well as data from the task. The study was approved by the ethics committee of the University Medical Center Utrecht.

### 2.2 Self-regulation Questionnaire

The full-scale Early Adolescent Temperament Questionnaire-Revised Short Form for parents (EATQ-R-SF: translated in Dutch by C.A. Hartman) is used to obtain a measure of self-regulatory capabilities (Ellis and Rothbart, 2001). This questionnaire was filled out by one of the parents on their child's behavior. Items on the 'inhibitory control' subscale were scored from a 1–5, where the final score was an average of all the items, with higher scores implying more inhibitory control. The mean score of the sample was 3.61 (SD = 0.54), with the values having a slight negative skew (−0.24) but normal distribution. Mean sample score was comparable to that of a sample of 1055 similarly aged children: 3.19 (SD = 0.50) (Muris and Meesters, 2009). As questionnaire data was not available from all subjects, Table 1 shows the number of subjects used in the analysis.

**Table 1:** Overview of the sample after exclusion of outliers, with a paired-samples *t*-test for sex differences.

	**Boys**	**Girls**	**Total**	**t**	**p**
**Participants** (n)	278	362	640		
**Age** in years, mean (sd)	9.51 (0.87)	9.50 (0.83)	9.50 (0.85)	0.11	0.91
**Righthanded** n	232 (83%)	322 (89%)	554 (87%)		
**EATQ-R** mean (sd), n	3.57 (0.55), 238	3.64 (0.52), 304	3.61 (0.54), 542	1.5	0.13
**PDS** mean (sd), n	1.15 (0.36), 208	1.38 (0.49), 260	1.28 (0.45), 468	5.5	< 0.0001

EATQ-R = Early Adolescent Temperament Questionnaire-Revised Short Form; PDS = Pubertal development questionnaire.

### 2.3 Pubertal development

The Pubertal development questionnaire (PDS) (Carskadon and Acebo, 1993) was used to get an indication of general levels of pubertal development in our sample. The cumulative score for girls (M = 5.4, SD = 1.8) was higher than for boys (M = 4.1, SD = 1.3), t(464) = 8.9, p < 0.001. As expected, the overall distribution of scores was positively skewed (1.71), with the majority of children falling in the pre- and early pubertal category (338 against 130).

### 2.4 Self-regulation functional MRI task

**Fig. 1:**
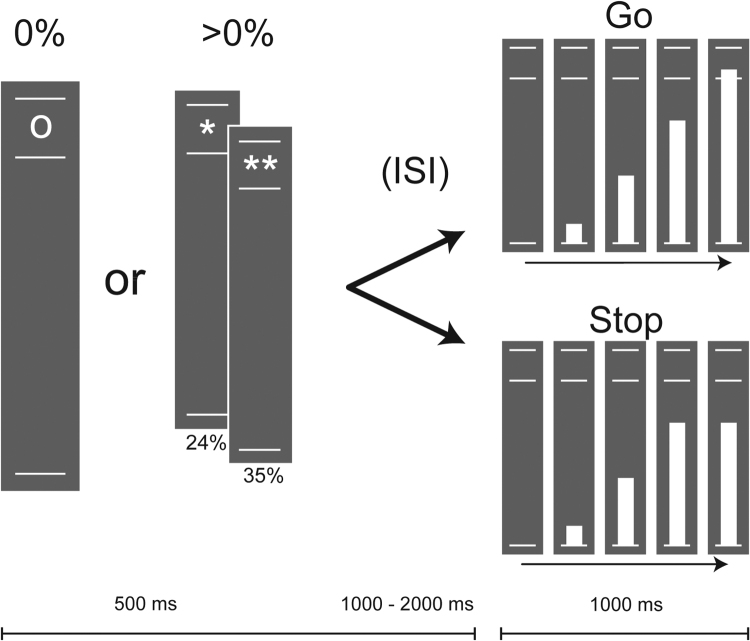
Stop signal anticipation task. Trials begin with the presentation of a cue (0, * or **), representing the stop-signal probability (0%, 22% and 33% respectively). Permanently visible are three horizontal white lines, goal is to stop a rising bar as close to the middle line as possible (target) by pressing a button, but refrain from pressing the button when the bar stops on its own (stop signal).

We will use behavioral measures and functional MRI data acquired while subjects perform the Stop-Signal Anticipation Task (SSAT) (more information on the YOUth Cohort study, see Onland*-*Moret et al., 2020). The SSAT provides us with several measures: inhibition speed and accuracy, identification of the regions associated with inhibitory control, a measure of relative activation in those regions, and the ability to measure functional coupling between those regions (Zandbelt and Vink, 2010), see Fig. 1. Subjects are presented with three parallel horizontal lines. On each trial, a bar moves at a constant speed from the lower line towards the upper line, reaching the middle line in 800 ms. The main task is to stop the bar as close to the middle line as possible, by pressing a button with the right thumb (i.e. Go trial). Stop trials are identical to Go trials, except that the bar stops moving automatically before reaching the middle line, indicating that a response has to be suppressed (i.e. stop-signal). The probability that such a stop-signal will appear is manipulated across trials and can be anticipated based on three different cues; '0' indicating 0%, '*' 22% and '**' 33% probability the bar will stop on its own. Task difficulty is adjusted to performance in a stepwise fashion, with a varying delay between the stop-signal and the target (i.e. the stop-line) depending on the success of the previous trial, thereby keeping the number of failed and successful trials comparable between subjects and sessions. This allows for a fair comparison between children that may possess varying levels of inhibitory control (Telzer et al., 2018). There were 256 trials in total presented in pseudorandom order: 85 trials with 0% probability, 86 trials with a 22% probability and 85 trials with a 33% probability. The initial order of trials was generated randomly once, and subsequently reused for each participant (trial sequence is included as a Supplemental Material).

### 2.5 Behavioral analysis

Reactive inhibition was measured by the latency (stop-signal response time; SSRT) and success of stopping on stop trials. The SSRT was computed according to the integration method (Logan and Cowan, 1984) and pooled across all stop-signal probability levels. This measure has been used as a behavioral indicator of inhibitory control (Fogel et al., 2019), and has been shown to be increased in children with inhibition problems, such as ADHD (Slusarek et al., 2001). Proactive inhibition was measured as the effect of stop-signal probability on go-signal response time. Adults subjects tend to slow down their responses as the probability of a stop becomes more likely (Vink et al., 2005). For all measures, the effect of age was estimated using a regression analysis with age as a continuous regressor and sex as a between-subject variable.

### 2.6 Image acquisition

The experiment was performed on a Philips (Philips medical systems, Best, the Netherlands) Ingenia 3.0 T MRI scanner at the UMC Utrecht. Functional images consisted of whole-brain, T2 * -weighted echo planar images with blood oxygen-dependent contrast [repetition time 1000 ms, echo time 25 ms, flip angle 65, 2.5 × 2.5 in-plane resolution, 2.5 mm slice thickness, 51 slices per volume, SENSE factor, 1.8 (anterior–posterior) and multiband factor 3] in a single run of 595 dynamic scans. A T1-weighted image from the same session was used for within-subject registration purposes.

### 2.7 fMRI analysis

#### 2.7.1 Preprocessing

Image data were processed using SPM12 (http://www.fil.ion.ucl.ac.uk/spm/). Preprocessing included realignment to correct for head motion, where the time-series were registered by a least-square approach and a rigid-body transformation. Then slice timing correction was applied by interpolating all slices in time to the center slice. Even with short repetition times and multiband, slice timing correction has been demonstrated to benefit results (Parker, 2019). Spatial normalization was done to the Montreal Neurological Institute template brain, and smoothing was applied (8 mm full width at half maximum) to correct for inter‐individual differences.

#### 2.7.2 Subject exclusion

Several subjects were excluded based solely on a fixed fMRI signal threshold, as the threshold for generating brain masks (default of 80% of global signal) can result in holes inside the mask for some subjects. This was due to either significant movement (possibly exacerbated by the multiband sequence), or general scanner artefacts. It is difficult to assess retrospectively whether signal artefacts are primarily due to scanner issues or motion artefacts. Subjects with voxels below the signal threshold within the brain, excluding cerebellum, were removed from the analysis. The total number of subjects excluded with this method was 151 (82 boys, mean age 9.6 years), leaving a dataset of 645 children (278 boys, mean age 9.5 years). During the subsequent analyses, 5 children were excluded from the dataset by being either an outlier in terms of signal (n = 2) or behavior (n = 3), leaving a final dataset of 640 children. There were 86 left-handed children in our sample, however all were instructed to perform the task using the right hand. As we did not have specific hypotheses on how handedness affects inhibitory motor control during the task, we did not exclude these participants from our analyses. We have rerun all analyses with right-handed children only to ensure that left-handedness did not change the significance of our main findings. These analyses are added as a Supplemental Material. See Table 1 for an overview of our sample, and Table 2 for an overview of the measures taken from the subjects.

**Table 2:** Overview of measures in the analysis.

**Measures**	**Type**	**Source**	**Description**
Self-regulation	Questionnaire	EATQ-R^1^	Score of self-regulation
Pubertal Development	Questionnaire	PDS^2^	Indication of pubertal development
SSRT	Behavior	Task	Reactive inhibition speed
Accuracy	Behavior	Task	Reactive inhibition accuracy
Response slowing	Behavior	Task	Proactive response slowing
ROI Brain activation	Neuroimaging	Task fMRI	Mean activation levels in predefined regions of interests during reactive and proactive inhibition
Psychophysiological interactions	Neuroimaging	Task fMRI	Measure of functional coupling between ROI regions during reactive and proactive inhibition

In this paper we will use the EATQ-R questionnaire as a measure of self-regulation in daily life. We will link this measure to measures of inhibitory control from the task and the associated brain measures.

1 The full-scale Early Adolescent Temperament Questionnaire-Revised Short Form for parents (EATQ-R-SF: translated in Dutch by C.A. Hartman) (Ellis and Rothbart, 2001).

2 Development scale questionnaire (PDS) (Carskadon and Acebo, 1993).

#### 2.7.3 Individual analyses

Functional images were submitted to a general linear model. Activation was time‐locked to the presentation of the cue and to the response period with a defined duration of 0 s, and was modeled using a hemodynamic response function based on stop‐signal probability. On average the inter‐trial interval was 1000 ms (ranging from 500 to 1500 ms), and served as an implicit baseline. Six realignment parameters were added as regressors of no interest to correct for residual signal changes related to head motion. All data were high‐pass filtered with a cut‐off of 128 s to control for low‐frequency drifts. For each participant, we computed two contrast images: (1) activation during successful stop trials versus failed stop trials (reactive inhibition), (2) activation during go trials with a stop-signal probability versus trials without (proactive inhibition). The first contrast is used to investigate the specific activation patterns associated with successful inhibition, based on previous research employing the same paradigm (Vink et al., 2014, Vink et al., 2015, Pas et al., 2017). The latter contrast is used to investigate the effect of proactive response slowing during the task, as the probability of a stop-signal appearing is expected to lead to the systematic slowing down of responses (Pas et al., 2019, Vink et al., 2015).

#### 2.7.4 Region of interest analyses

The two contrast images per subject were subjected to a one-sample *t*-test group-level analysis, resulting in two group-level brain maps. To determine effects of sex and age on activation in these contrasts, regression analyses were performed on predefined regions of interest (ROIs), created using the MarsBaR toolbox (http://marsbar.sourceforge.net). For proactive inhibition the regions are based on the activation patterns in young adults from Pas et al. (2019), resulting in three regions; the right mid frontal cortex, the right parietal cortex and the right putamen. The reactive inhibition ROIs were based on activation in young adults from Pas et al. (2017), and consisted of the left motor cortex, and the bilateral striatum. From these ROIs, we extracted the mean activation level for each participant for the two contrasts of interest. A one-sample *t*-test was used to test for significant activation in the selected ROI for the two contrasts. Mean activation levels of all ROI were subsequently subjected to a regression analysis with age as continuous predictor, and sex as a between-subject variable. No additional manipulations or corrections were performed on the independent variables. The statistical threshold for significance was set at p = 0.05/3 = 0.017, to correct for multiple comparisons (Bonferroni correction with three ROIs per contrast). Figures of the ROIs for proactive and reactive inhibition are included in the supplemental materials.

#### 2.7.5 Functional coupling

Functional coupling analyses were performed using psychophysiological interaction (PPI) (Friston et al., 1997) to investigate the effect of age and sex on the coupling between ROIs of the frontostriatal network. These measures serve as an indication of similarity in activation of two different regions in the brain. A similar analysis using fMRI data and the same task has been performed before by Vink and colleagues (2014), which found significant frontostriatal functional coupling during proactive inhibition in an adolescent sample. We therefore opted to employ the same seed region; a 6-mm-radius sphere around the center-of-mass of the right striatum (MNI coordinates [20, 12, 0]). A PPI analysis was performed to investigate the functional coupling on the proactive inhibition contrast, go trials with a stop-signal probability versus trials without. Coupling was investigated between the seed and the right mid frontal cortex, and the parietal cortex. For reactive inhibition, functional coupling was investigated during successful stop trials versus unsuccessful stop trials (i.e., psychological factor) between the seed and the left motor cortex. For each participant, the first eigenvariate of the BOLD signal for the seed region was calculated and adjusted for average activation during the task and head motion. The interaction between activity within the seed region and the psychological factor was then calculated, for both positive as well as negative relationships. The resulting individual contrast images were entered into a second-level analyses to test for the effect of sex and age on functional coupling.

#### 2.7.6 Group analysis

To investigate potential activation patterns outside the predefined ROIs, an additional whole-brain group analysis was conducted on the contrasts defined in Section 2.7.3 'Individual analyses'. We employed significance testing using voxel-wise inference. Due to our large sample size, we opted for a FWE (Bonferroni) correction for multiple testing at the voxel level, p < 0.05.

## 3 Results

### 3.1 Confounds

First, we tested for age and sex-related effects on motion during the task using a regression analysis. This revealed no main effect of sex F(1, 633) = 0.73, p = 0.39, but did show an effect of age **F(1, 636) = 19.37, p < 0.001**, with motion being significantly lower in older than younger children. We performed additional Pearson's correlation tests to see whether movement during the task was related to our behavioral measures of reactive and proactive inhibition, or scores of self-regulation. Such an association was found in a previous fMRI study investigating inhibitory control (Stange et al., 2018), and this would present a possible confound for our current study. However, the resulting correlation coefficients ranged from −0.06–0.13, and none were significant. The tests were also run with the excluded subjects re-added to the sample, but the correlation coefficient ranged from −0.08–0.10, and none were significant.

### 3.2 Behavior

We then assessed effects of sex and age on reactive and proactive behavioral measures from the task. We found that reactive inhibition latency (SSRT) was significantly associated with age **F(1, 636) = 104.84, p < 0.001** (r = −0.38), but not with sex F(1, 637) = 3.08, p = 0.08, indicating that older children where faster at inhibiting responses than younger children. Inhibition accuracy also improved with age **F(1, 636) = 26.67, p < 0.001**, regardless of sex F(1, 636) = 1.00, p = 0.32. Slowing down responses in anticipation of a stop-signal is a measure of proactive inhibition. Participants slowed their responses more with increasing stop-signal probability, **F(2, 638) = 248.71; p < 0.001**, regardless of sex, F(2, 638) = 2.52, p = 0.11. This proactive response slowing was not associated with age F(1, 636) = 2.97, p = 0.09, nor with sex F(1, 637) = 0.48, p = 0.49. While older children were both significantly faster and more accurate in response inhibition than younger children, they did not show an increase in response slowing.

**Fig. 2:**
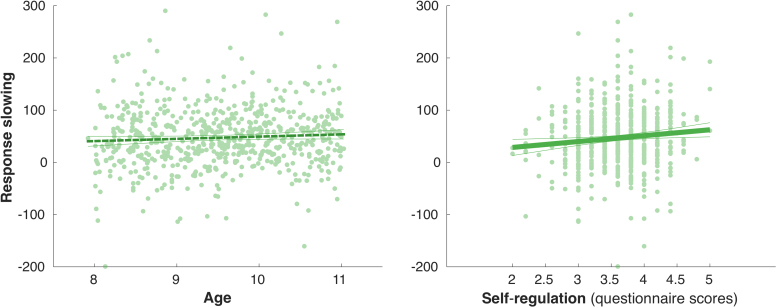
Proactive response slowing against age and self-regulation scores. LEFT: Scatter plots of response slowing on the task plotted against age, not significant; RIGHT: scores on self-regulation as measured by the EATQ-R questionnaire as a function of age (with linear trend line and 95% confidence interval)*.*

The 'inhibitory-control scale' of the EATQ-R served as a proxy of more general self-regulation abilities of the children. There was no effect of age on the scores, F(1, 536) = 2.57, p = 0.11; nor sex, F(1, 536) = 2.59, p = 0.11; nor an interaction effect, F(1, 536) = 0.92, p = 0.34. To test our hypothesis that children scoring higher on this scale show better inhibitory control during the task, a regression analysis was conducted for reactive and proactive measures of inhibition. There was no significant main effect of the scores on SSRT (e.g. reactive inhibition), F(1, 536) = 0.43, p = 0.51. However, we did find a significant relation between self-regulation and proactive response slowing on the task, **F(1, 533) = 6.48, p = 0.01**, regardless of sex F(1, 533) = 0.54, p = 0.46 (Fig. 2).

### 3.3 Activation

#### 3.3.1 Region of interests

A one-sample *t*-test was used to test for significant activation in the selected ROI. For reactive inhibition we found deactivation in the left motor cortex in the contrast of successful stop trials versus failed stop trials**, t(639) = −2.39, p = 0.02**, indicating suppression of the motor cortex during successful inhibition. However, this result does not survive correction for multiple comparisons (Bonferroni with p < 0.05/3 = 0.017). In addition, there was significant bilateral activation in the striatum, left **t(639) = 10.67, p < 0.001**, and right **t(639) = 10.65, p < 0.001**. For proactive inhibition, there was significant activation in the network associated with proactive inhibition: the mid frontal cortex, **t(639) = 9.11; p < 0.001**, the right parietal cortex, **t(639) = 6.90; p < 0.001**, and the right putamen **t(639) = 4.42; p < 0.001**. These results survive correction for multiple comparisons (p < 0.05/3 = 0.017). A linear regression analysis with age as a continuous variable and sex as a within subject variable yielded no significant effects, see the Supplemental Materials for a detailed analysis.

**Fig. 3:**
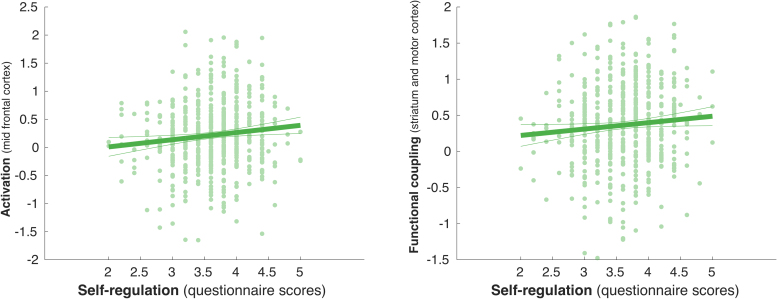
Self-regulation scores against brain activation and functional coupling. Scatter plot of activation in the mid frontal cortex during proactive inhibition (see ROI results in Section 3.3.1; see Supplementary Materials Figs. 5 and 6 for a visualization of the ROIs) against self-regulation questionnaire scores (LEFT) and functional coupling between the right striatum and left motor cortex during reactive inhibition as a function of self-regulation questionnaire scores (RIGHT) (with linear trend line and 95% confidence interval).

We expected children scoring higher on self-regulation to exhibit more activation in the frontal cortex. Using a linear regression analysis, we found that activation in the right mid frontal cortex during proactive inhibition was associated with self-regulation**, F(1, 536) = 6.37, p = 0.01 (r = 0.11)**, with no interaction effect for sex F(1, 536) = 0.07, p = 0.79 (corrected for multiple comparisons for the three ROI, with p < 0.05 / 3 = 0.016) (Fig. 3).

#### 3.3.2 Functional coupling

To investigate the degree to which areas of the brain are functionally connected, we tested for effects of sex and age on functional coupling between cortical and subcortical ROI of the brain. During reactive inhibition, there was an effect of age on functional coupling between the right striatum and the left motor cortex**, F(1, 636) = 4.93, p = 0.03**. There was no effect of sex, F(1, 633) = 1.71, p = 0.19, though there was an interaction effect between sex and age, **F(1, 636) = 4.66, p = 0.03**. A post-hoc regression analysis revealed that this association with age, was specifically present for girls **F(1, 361) = 10.52, p < 0.01 (r = 0.17)**, but not for boys F(1, 277) = 0.01, p = 0.92 (r = 0.01); (Bonferroni corrected for multiple comparisons at p < 0.05/2 = 0.025). An additional analysis splitting the two sexes in groups based on pubertal development, revealed that boys with mid pubertal characteristics had significantly more coupling between the two ROI, than boys in pre- and early puberty **t(205) = 2.95, p < 0.01**. There was no such difference for girls.

During proactive inhibition, regression analyses showed that activation in the striatum was more strongly coupled with the mid frontal cortex for older than younger children, **F(1, 636) = 7.21, p = 0.01**. There was no effect for sex F(1, 633) = 2.13, p = 0.14, but there was an interaction effect between sex and age, F(1, 636) = 10.26, p < 0.001. A post-hoc regression analysis revealed that the association with age was specifically present for boys **F(1, 277) = 15.56, p < 0.001 (r = 0.23)**, but not for girls F(1, 361) = 0.03, p = 0.85 (r = −0.01); (Bonferroni corrected for multiple comparisons at p < 0.05/2 = 0.025). There was no difference in coupling based on pubertal development, for either sex. For the right parietal cortex, there were no effects of age and sex altogether.

Our final hypothesis was that increases in self-regulation would be paralleled by more functional coupling between subcortical and cortical areas of the brain. For reactive inhibition, using a regression analysis, we found a moderate relationship of self-regulation questionnaire scores and coupling between the left motor cortex and the right striatum, **F(1, 536) = 4.18, p = 0.04**; but no interaction with sex, F(1, 536) = 3.08, p = 0.08 (Fig. 3). There was no effect of the scores on frontostriatal coupling during proactive inhibition, F(1, 536) = 0.81, p = 0.37; and no interaction with sex, F(1, 536) = 1.34, p = 0.25.

#### 3.3.3 Whole brain

**Fig. 4:**
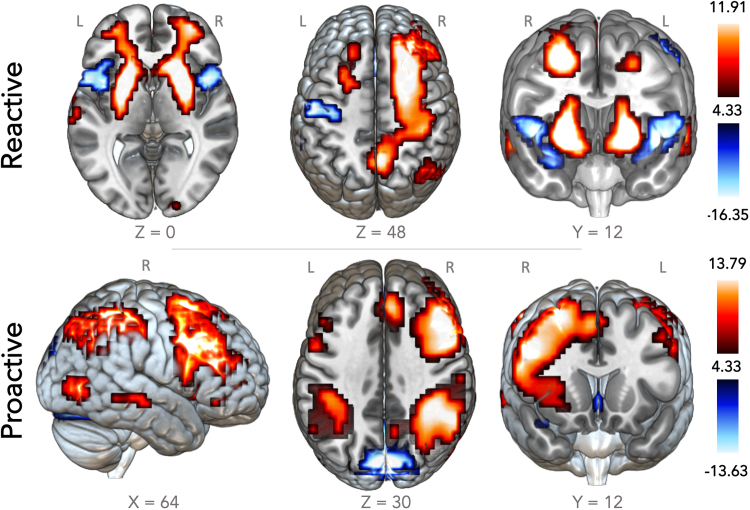
Significant activation clusters during reactive and proactive inhibition. Above 'Reactive inhibition': correct versus incorrect stop trials. Significant clusters of positive activation (more activation during correct compared to incorrect trials) include the bilateral putamen, superior temporal gyrus and the precuneus. Deactivation (lower activation during correct compared to incorrect trials) occurred in the bilateral cerebellum, cingulate and postcentral gyrus and the left insula. Below 'Proactive inhibition': Go trials with > 0% stop-signal probability versus 0%. Significant clusters of positive activation (more activation during >0% stop-signal probability compared to 0%) include the right mid frontal cortex, the right parietal cortex and the right putamen. Deactivation (lower activation during trials with a >0% stop-signal probability compared to 0%) was found bilaterally in the cuneus and the anterior cingulate cortices (FWE corrected at p < 0.05, height threshold T = 4.33). The group maps are available for viewing on NeuroVault: https://neurovault.org/collections/XHYBGZPM/.

To explore brain regions associated with reactive and proactive response inhibition outside of our predefined regions of interest, two whole brain analyses were conducted. During reactive inhibition we found significant clusters of activation in the bilateral putamen, superior temporal gyrus and the precuneus. Deactivation was found in the cerebellum, cingulate and postcentral gyrus left insula. For proactive inhibition we found significant activation clusters in the right mid. Frontal gyrus extending into the putamen, cerebellum, bilateral inferior parietal lobes and bilateral temporal gyri, with significant deactivation in the cuneus and anterior cingulate. See Fig. 4 and Table 3 for an overview of activation clusters. An extra figure for illustrative purposes was added as a Supplemental Material that shows our predefined ROIs displayed on top of our whole-brain results.

**Table 3:** Overview of activations.

**Region**	**BA**	**Side**	**No. of voxels**	**X**	**Y**	**Z**	**Max t-value**
**Reactive inhibition**							
***Positive***							
Putamen		L	347	-24	8	-4	13.79
Putamen		R	1618	24	12	0	13.66
Superior Temporal Gyrus	22	L	75	-64	16	0	6.46
Precuneus	31	L	83	-24	-36	28	5.84
***Negative***							
Cerebellum		L	379	-32	-52	-20	13.63
Cerebellum		R	264	-32	-56	-16	12.22
Cingulate Gyrus	32	L/R	89	0	24	28	8.40
Postcentral gyrus	1	L	60	-52	16	-20	8.19
Insula	13	L	141	-44	8	-4	6.79
**Proactive Inhibition**							
***Positive***							
Mid. frontal gyrus	9	R	1183	48	36	24	10.15
Inf. Parietal lobe	40	R	692	48	-44	52	11.94
Inf. Parietal Lobe	40	L	615	-44	-36	44	8.86
Cerebellum		L/R	483	-8	-80	-32	8.48
Mid. Temporal gyrus	37	R	139	48	-68	0	8.75
Mid. Temporal gyrus	37	L	72	-44	-72	4	8.52
***Negative***							
Cuneus	18	L/R	1869	8	-92	24	16.41
Anterior Cingulate	24	L/R	155	0	28	-8	7.37

All results are significant at a voxelwise FWE correction of p < 0.05, height threshold T = 4.33; L, left; R, right; X Y Z refer to the center of mass with labels taken from the MNI atlas (nearest grey matter).

## 4 Discussion

In this study, we investigated whether self-regulatory abilities in children are reflected in brain measures. We present data on the relationship between self-regulation and neural correlates of reactive (i.e., outright stopping), and proactive inhibition (i.e., anticipation of stopping) in a cohort of 640 healthy children aged 8–11 years. Behaviorally, we find that even in the narrow age-range spanning 3 years, there are advances in inhibitory control speed and accuracy. Both boys and girls slowed down their responses in anticipation of a stop, demonstrating that proactive inhibitory control is already present. Notably, we found that an independent measure of self-regulation was associated with the amount of proactive response slowing on the task. In the brain, we found significant activation in brain regions associated with reactive and proactive inhibition. During reactive inhibition, there was increased activation in the bilateral striatum. During proactive inhibition, there was increased activation in the right mid frontal gyrus, the right inferior parietal lobe and the right putamen. Activation in these regions was not associated with age and did not vary between boys and girls. However, self-regulation scores were positively associated with activation in the frontal cortex during proactive inhibition. Finally, we found several age-related changes that differed between the sexes. In girls, functional coupling between the right striatum and the left motor cortex increased with age during reactive inhibition. In boys, fronto-striatal functional coupling (between the right striatum and the mid frontal cortex) increased with age during proactive inhibition. In our sample, reactive inhibition improved significantly in terms of speed and accuracy in the span of three years. Both older boys and girls are more skilled at inhibiting responses than their younger counterparts, in line with other studies (Bedard et al., 2002, Durston et al., 2002, Tamm et al., 2002, Rubia et al., 2013, Velanova et al., 2009, Van de Laar, 2011). Notably, our data shows that even young children aged 8–11 years already exhibit proactive response slowing. This effect of responses becoming slower with increasing stop-signal probability has been consistently established in adults (Vink et al., 2005; Chikazoe et al., 2009; Verbruggen and Logan, 2009; Jahfari et al., 2010; Zandbelt and Vink, 2010; Vink et al., 2015; Pas et al., 2019, Pas et al., 2017), with some evidence showing that adolescents also exhibit this feature (Vink et al., 2014). Where recent studies have looked at proactive inhibitory control in younger children in terms of performance monitoring (Hadley et al., 2019), or differences in proactive inhibition between ADHD and healthy control children (van Hulst et al., 2018) - our study is the first to investigate sex and developmental effects on both reactive and proactive inhibition in a sample of children at a young age. Bilateral activation of the striatum was associated with reactive inhibition. This region has been consistently associated with the suppression of motor responses (Vink et al., 2005, Aron and Poldrack, 2006, Zandbelt and Vink, 2010), and modulating the response threshold (Lo and Wang, 2006, Forstmann et al., 2008, Jahfari et al., 2010). Previous research links striatal activation during reactive inhibition to the prior anticipatory processing of contextual cues (Vink et al., 2015, Pas et al., 2017). This makes it difficult to pin-down its specific role, where effects stemming from formed expectations and successful performance on the task may intertwine. The level of activation was not associated with age, nor did it differ for the two sexes. Some studies have pointed to a decrease in striatal activation with age during reactive inhibition (Casey et al., 1997, Durston et al., 2002, Rubia et al., 2007). A previous study with a sample of adolescents also failed to find an association with age – albeit in a much smaller sample (Vink et al., 2014). It may be that a decrease in striatal activation during reactive inhibition is paralleled by an increase during proactive inhibition, but that this shift relies on the relative maturation of frontostriatal networks. However, conducting such an analysis was not possible using the current dataset. The shift may be similar to the temporal shift in striatal activation from reward receival to the anticipation of the reward (Schultz*,* 1997). When rewards can be predicted by a cue, striatal activation increases in anticipation and less as a reaction to receiving of the reward. Previous research has shown that this shift develops throughout adolescence (Bjork et al., 2010, Hoogendam et al., 2013, Vink et al., 2014). This is supported by research showing that the striatum is associated with the learning of stimulus-response associations, but not with their application (Vink et al., 2013). Next to activation in our predefined regions of interest, our whole-brain analyses revealed additional brain areas where significant activation occurred. These activation patterns are in line with literature on response inhibition and motor control, specifically for the Superior Temporal Gyrus (Horn et al., 2003), and the Precuneus (Wenderoth et al., 2005). We also found significant deactivation of the bilateral insula, implicated in motor preparation (Hester et al., 2004). Functional coupling between the left motor cortex and the right striatum increased with age, specifically for girls. Among boys, those further along in pubertal development also exhibited more functional coupling. This is in line with previous research showing a positive association between functional coupling and age in adolescents (Vink et al., 2014). This difference for the two sexes points to possible distinct developmental trajectories. It may be that boys already show higher levels of coupling at a younger age and therefore have less room for increases, although this difference was not significantly present in our sample.

During proactive inhibition, children in our sample predominantly exhibited activation in cortical areas such as the right parietal cortex and right mid frontal cortex, with the activation cluster extending into the striatum. Response inhibition studies have commonly reported an association between striatal activation and the anticipation of stop‐signals (Aron and Poldrack, 2006, Hu and Li, 2012, Vink et al., 2005, Vink et al., 2015, Zandbelt and Vink, 2010). The broader area of the basal ganglia has been hypothesized to act as a gatekeeper, preventing execution of conflicting motor responses (Friend and Kravitz, 2014, Mink, 1996), and incorporating prior reinforcement (Vink et al., 2013). In addition to the striatum, the right mid frontal cortex has long been recognized as playing an important role in proactive inhibition (Aron et al., 2003, Rubia et al., 2003; Vink, et al., 2015). An increase in functional connectivity between this area and the basal ganglia has been shown to increase response inhibition efficiency (Xu et al., 2016). In contrast, hypoactivation of the right frontal cortex in patients with ADHD has been linked to impaired response inhibition (Morein*-*Zamir et al., 2014). The largest cluster of activation during proactive inhibition was present in the right parietal cortex. Activity in this area has been linked to self‐initiated as opposed to triggered or automatic responses (Kühn et al., 2008), the storage of acquired motor skills (Halsband et al., 2001, Niessen et al., 2014) , involvement in response selection (Dippel and Beste, 2015). The parietal cortex and mid temporal gyrus were found to be bilaterally activated, with a large cluster of deactivation centered around the cuneus. Deactivation of the cuneus has previously been found during go/no-go tasks. One theory is that this deactivation may resemble a task demand sensitive cross-modal inhibition mechanism that optimizes performance by reducing potentially distracting neural processes (Laurienti et al., 2002, Talanow et al., 2020).

We saw a significant association with age and functional coupling of the right striatum and the right mid frontal cortex, specifically for boys. Previous research has shown increases in functional connectivity between these regions in an older sample of children (Vink et al., 2014). During adolescence, maturation of brain regions varies spatiotemporally over the brain, with subcortical regions related to motivation maturing before prefrontal development (Casey, 2015, Casey et al., 2008, Gladwin et al., 2011). Our data shows that a degree of variability exists between the sexes regarding functional coupling, though it is not clear whether these differences will persist throughout development or are temporary. Sex differences have been found in brain volume, with a larger increase in white matter for males compared than females (Giedd et al., 1999, De Bellis et al., 2001, Lenroot and Giedd, 2010). Research into sex differences in the brain during inhibition has also pointed to differences in frontostriatal activation (Rubia et al., 2013).

Our aim was to determine whether children who show high levels of self-regulation also show high levels of reactive and proactive inhibitory control. We found that children with higher self-regulation scores demonstrated more response slowing during the task. It is presumed that the improvement in self-regulation in adolescence is in part due to the effective integration and coordination of executive functions, leading to the rise of proactive response strategies that allow for a more efficient processing by engaging inhibitory functions prior to having to inhibit responses (Zandbelt and Vink, 2010, Vink et al., 2020). In terms of brain activation, self-regulation was positively associated with activation in the right mid frontal cortex during proactive inhibition. This finding is in line with the notion that proactive inhibitory control relies on the establishment of frontal control over the rest of the brain, in particular subcortical regions (Cools, 2011, Vink et al., 2014). The right frontal cortex has long been recognized as playing an important role in proactive inhibition (Aron et al., 2003, Rubia et al., 2003, Vink et al., 2015, Pas et al., 2019). Finally, self-regulation scores were also correlated with functional coupling between the right striatum and the left motor cortex. On the one hand functional coupling between these two regions may point to an increase in efficiency of motor inhibition, and that this is reflected in the general ability of inhibitory control in daily life. For instance, the ability to suppress automatically elicited responses may help in controlling eating behavior (Fogel et al., 2019). Alternatively, the increase in functional coupling during reactive inhibition may not be limited to our selected ROI, and reflect a more general trend of increasing connectivity between subcortical and cortical structures (Duijvenvoorde et al., 2019).

### 4.1 Limitations

A number of limitations need to be considered. First, our results are based on an fMRI paradigm in children. In an adult sample this specific task has a moderate reliability (Buimer et al., 2020), and data from children will generally be more confounded due to issues of head motion or task compliance (Greene et al., 2018). We chose to employ strict objective parameters for subject exclusion, resulting in 151 children being left out of our analysis. While our remaining sample size was large enough to test our main hypotheses, we lack the power to reliably investigate individual differences and must stick to general group characteristics. In addition, the fact that we did not have questionnaire data from all children results in smaller subgroups.

Head motion can produce spurious signal fluctuations that may confound measures of functional coupling (Ciric et al., 2018). While we have taken measures of reducing head motion issues, some residual effects will remain present in the data. Due to the head moving from a fixed origin (the neck) the strength of short‐range connections can increase as they are more similar in their timing of movement, as opposed to long-range connections that become weaker (Satterthwaite et al., 2014). In terms of our functional coupling results, the main effects were significantly different between the sexes whereas movement did not differ.

### 4.2 Conclusion

Our data shows for the first time in children, that self-regulation is related to behavioral and neural correlates of inhibitory control. First, we showed that even children at a young age exhibit proactive inhibitory control over their actions, while reactive inhibition improved with age. Children scoring higher on self-regulation demonstrated more proactive inhibitory control in terms of slowing down their responses, higher activation in the mid frontal cortex and more functional coupling between subcortical and cortical areas. This paper does not provide a definitive answer to how increases in self-regulation during childhood relate to changes on a neural level, however, this cross-sectional data does shed light on several neural correlates that may be of importance in development. The associations between self-regulation and neural underpinnings in our sample undoubtably are limited in size, as such this data may benefit from optimizing methods of reducing noise that may be present in both questionnaire and brain data. When cohort data from a second wave will be made available, future research can employ longitudinal designs to further investigate the neural aspects of self-regulation. In theory, the state of self-regulation in the brain at a young age could subsequently be used to make predictions on well-being, school results and drug usage in adolescence.

## Data statement

Data is accessible under restriction as outlined in the YOUth Data Access Protocol on the data access page of the YOUth website (https://www.uu.nl/en/research/youth-cohort-study/data-access). Access to the data used in the current publication for verification purposes is possible, and requests should be directed at the corresponding author.

## Declaration of Competing Interest

The authors declare that they have no known competing financial interests or personal relationships that could have appeared to influence the work reported in this paper.
