# Supplementary material for: Self-regulation in the pre-adolescent brain
Source: Dev Cogn Neurosci. 2021 Sep 10;51:101012. doi: 10.1016/j.dcn.2021.101012 (PMC8450202; doi:10.1016/j.dcn.2021.101012)
Supplement: Supplementary file 1 — Supplementary material [file mmc1.docx]

**SUPPLEMENTAL MATERIALS**

**S.1 Supplemental figures**

**
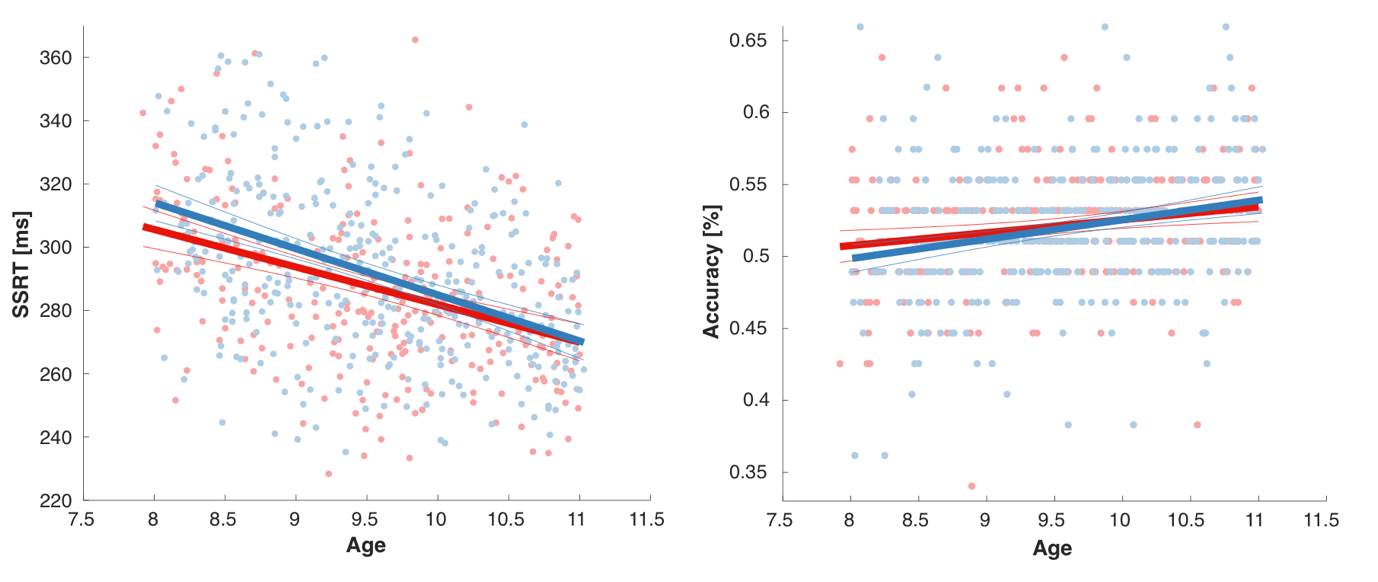
**

**Figure 1**. Behavioral measures of reactive inhibition. Data for response latency (SSRT) on the left, and inhibition accuracy on the right, for boys (red) and girls plotted against subject age (with linear trend line and 95% confidence interval).


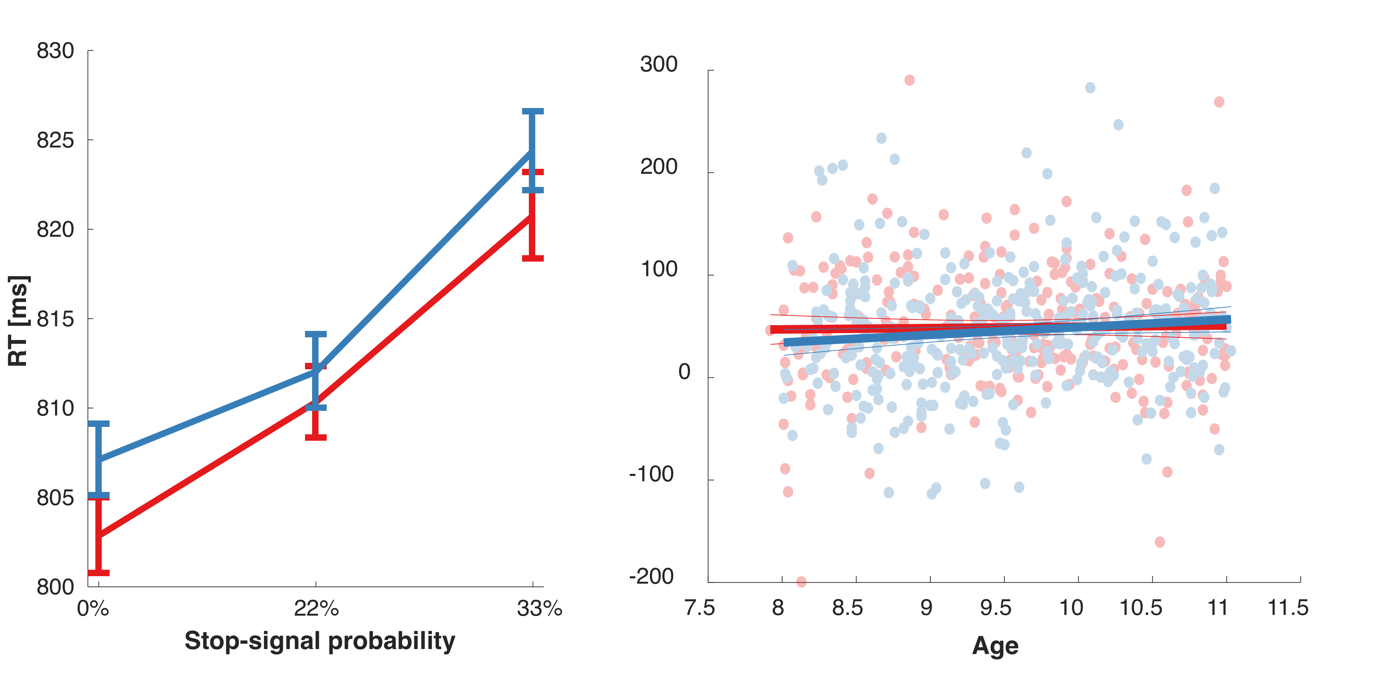


**Figure 2.** Behavioral measures of proactive inhibition. On the left response time for boys (red) and girls during each stop-signal probability condition. On the right the amount of response slowing as a function of age.

**
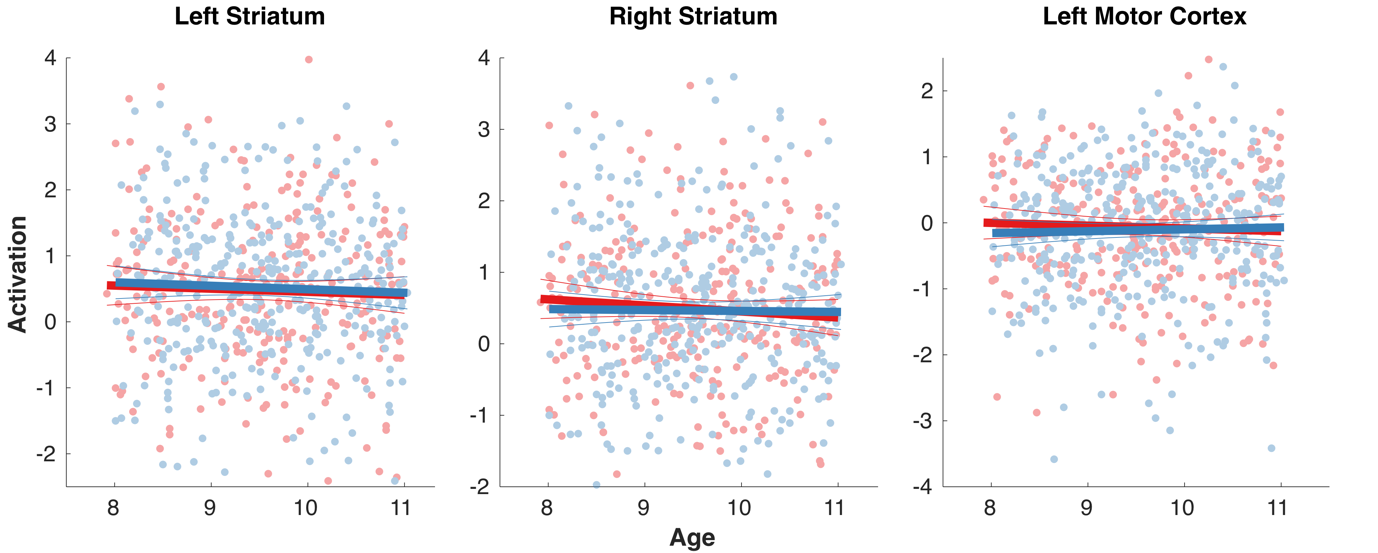
**

**Figure 3. Mean activation levels for reactive inhibition per ROI.** Scatter plots (regression coefficients) as a function of age (with linear trend line and 95% confidence interval)**.**

**
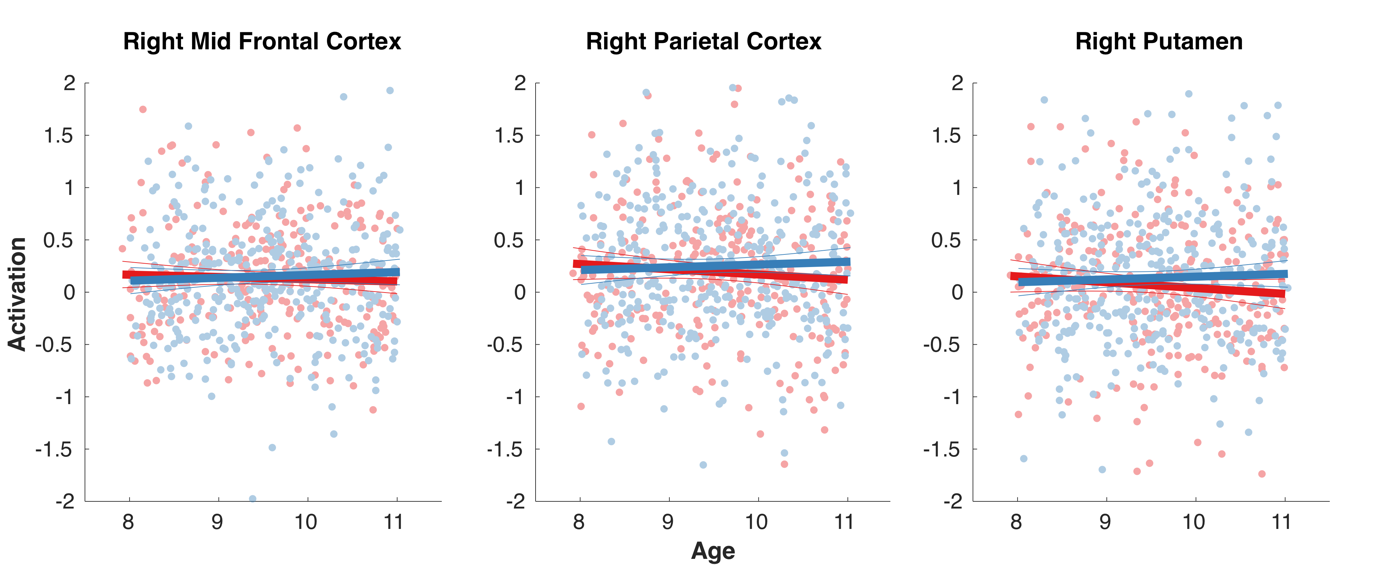
Figure 4. Mean activation levels for proactive inhibition per ROI.** Scatter plots (regression coefficients) as a function of age (with linear trend line and 95% confidence interval)**.**

***
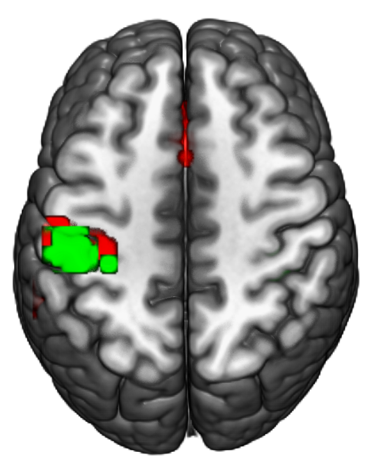

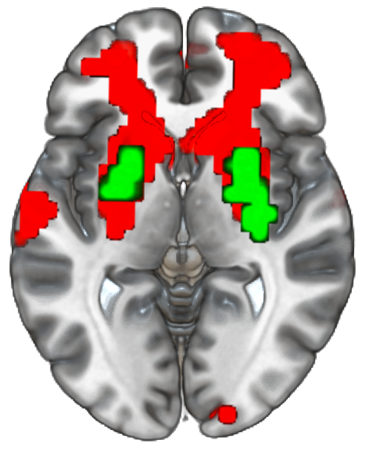

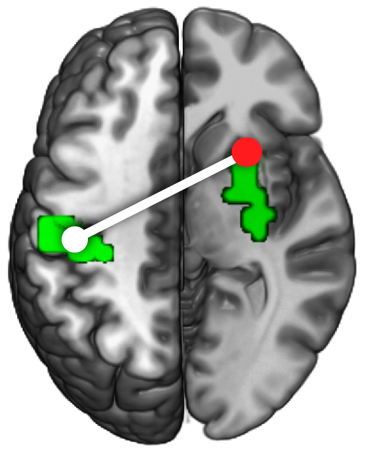
***

**Figure 5. ROI for reactive inhibition.** On the left shown in green the ROI of the left-motor cortex on top of the whole-brain deactivation pattern in red, middle the bilateral striatum ROI in green on top of the activation pattern - of the current sample when contrasting correct versus incorrect stop trials. On the right image the connection is visualized that we test in the functional coupling analysis, between the left motor cortex and the right striatum with in red the PPI VOI seed.


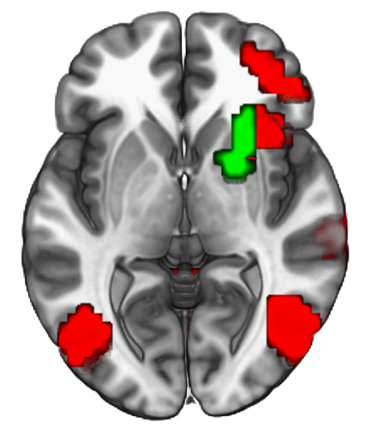

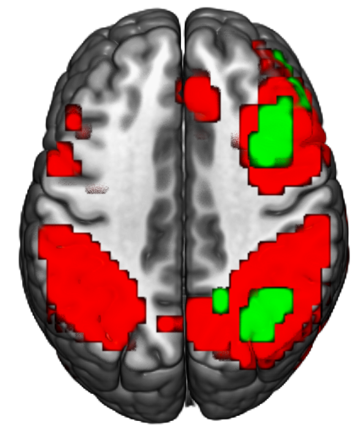

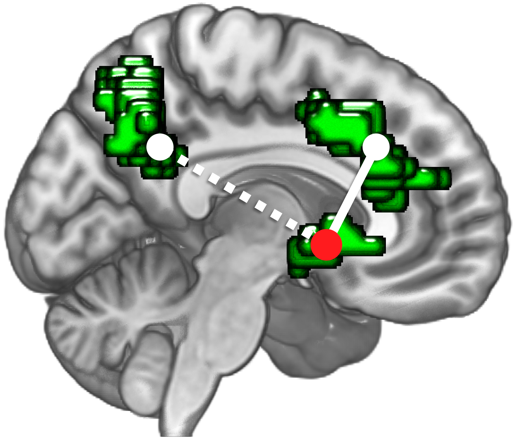


**Figure 6. ROI for proactive inhibition.** The two images on the left show In green the ROI on top of the whole-brain activation pattern of the current sample when contrasting Go trials with >0% stop-signal probability versus 0%. These are the mid frontal cortex, the right parietal cortex and the right putamen. On the rightmost image the two connections are visualized that we test in the functional coupling analysis, frontostriatal (solid) and between the striatum and parietal cortex (dotted), with in red the PPI VOI seed.


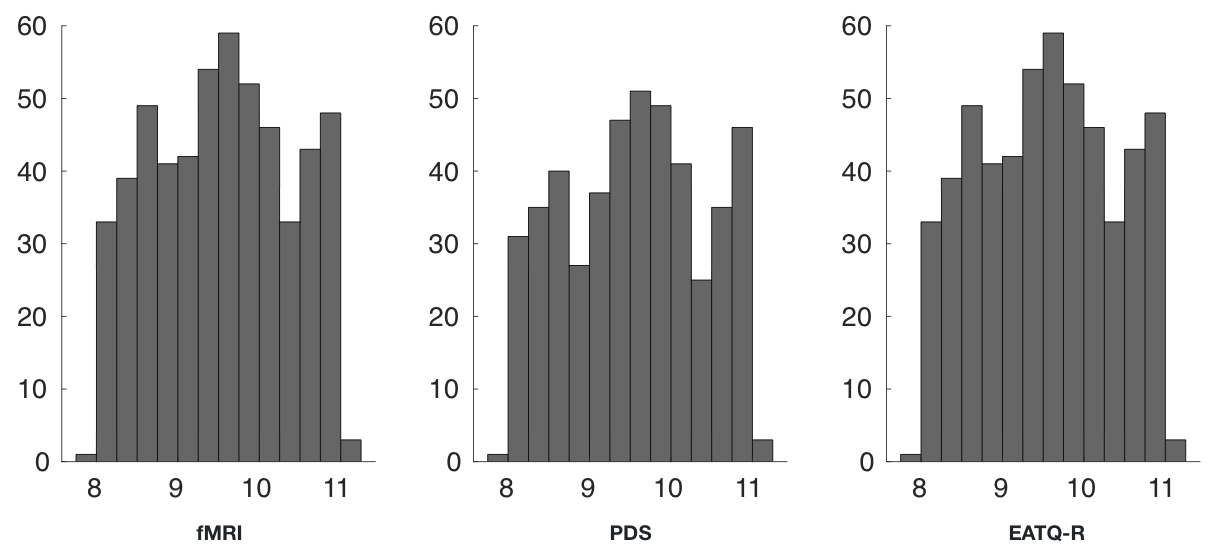


**Figure 7. Age distributions for the three measures.** Left the distribution for the fMRI sample (n = 645), in the middle the sample for which a Pubertal Development Scale was available (n = 542) and on the right the sample for which the EATQ-R was administered (n = 468).

**S.2 ROI analyses**Subsequent regression analyses for both proactive and reactive inhibition showed that this activation was not associated with age nor sex. Specifically, for the right mid frontal cortex, there was no effect of sex F(1,633) = 0.81, p = 0.37, age F(1,633) = 0.10, p = 0.75 (r = -0.01), nor an interaction F(1,637) = 2.02, p = 0.16. For the right parietal cortex there again were no effects of sex F(1,637) = 1.63, p = 0.20, age F(1,633) = 0.23, p = 0.63 (r = -0.02), nor an interaction F(1,637) = 1.49, p = 0.22. Finally for the right putamen, there again was no main effect for sex F(1,637) = 4.37, p = 0.14, and age F(1,633) = 0.24, p = 0.63 (r = -0.02), nor an interaction effect F(1,637) = 0.69, p = 0.41. Statistical threshold set at p < 0.05/3 = 0.017 per contrast.

**S.3 Functional Coupling**

The results from the PPI analyses are presented in figure x. During reactive inhibition, there was an effect of age on functional coupling between the right striatum and the left motor cortex, F(1,636) = 4.93, p = 0.03. There was no effect of sex, F(1,633) = 1.71, p = 0.19, though there was an interaction effect, F(1,636) = 4.66, p = 0.03. A post-hoc regression analysis revealed that this association with age, was specifically present for girls F(1,361) = 10.52, p < 0.01 (r = 0.17), but not for boys F(1,277) = 0.01, p = 0.92 (r = 0.01); (Bonferroni corrected for multiple comparisons at p < 0.05/2 = 0.025). An additional analysis splitting the two sexes in groups based on pubertal development, revealed that boys with mid pubertal characteristics had significantly more coupling between the two ROI, than boys in pre- and early puberty t(205) = 2.95, p < 0.01. There was no such difference for girls.

During proactive inhibition, regression analyses showed that across development activation in the striatum became more strongly coupled with the mid frontal cortex, F(1,636) = 7.21, p = 0.01. There was no effect for sex F(1,633) = 2.13, p = 0.14, but there was an interaction effect, F(1,636) = 10.26, p < 0.001. A post-hoc regression analysis revealed that the association with age was specifically present for boys F(1,277) = 15.56, p < 0.001 (r = 0.23), but not for girls F(1,361) = 0.03, p = 0.85 (r = -0.01); (Bonferroni corrected for multiple comparisons at p < 0.05/2 = 0.025). There was no difference in coupling based on pubertal development, for either sex. For the right parietal cortex, there were no effects of age and sex altogether.

**
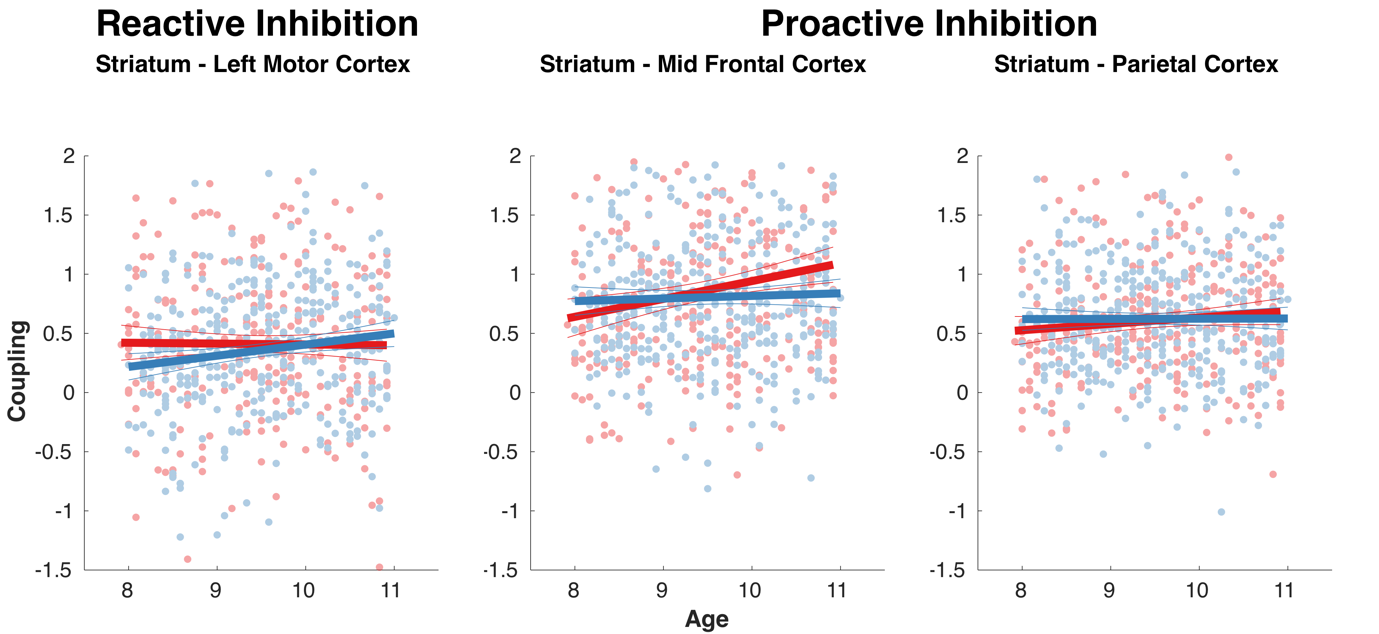
**

**Figure 7.** Scatter plots of the level of functional coupling (regression coefficients) for the left-motor cortex (LEFT) during reactive inhibition, and right mid frontal cortex (MIDDLE) and the right parietal cortex (RIGHT) during proactive inhibition, with the right striatum as a seed, plotted against age (with linear trend line and 95% confidence interval), for boys (red) and girls.

For reactive inhibition, there also was a relationship between SSRT (measurement of reactive inhibition speed) and coupling between the left motor cortex and the right striatum, F(1,636) = 8.04, p < 0.001; but no interaction with sex, F(1,636) = 0.00, p = 0.97. There was no association between response slowing on the task (measurement of proactive inhibition) and frontostriatal coupling, F(1,636) = 1.42, p = 0.23; nor an interaction with sex, F(1,636) = 0.56, p = 0.46.

**S.7 Handedness**Our main regression analyses were rerun with all left-handed children excluded. The relationship between self-regulation and proactive response slowing on the task remained significant, F(1,459) = 6.23, p = 0.01, regardless of sex F(1,459) = 0.49, p = 0.48. The relationship between self-regulation and activation in the frontal cortex remained significant as well, with F(1,459) = 4.51, p = 0.03, regardless of sex, F(1,459) = 0.20, p = 0.65. Then finally the relationship between self-regulation and functional coupling. This association remained significant as well with F(1,459) = 3.50, p = 0.03, with no effect of sex, F(1,459) = 2.51, p = 0.08.

**S.8 Additional information on whole-brain analysis**

For the whole-brain analyses the estimated FWHM by SPM12 was 20.2mm x 19.1mm x 15.4mm (5.1x4.8x3.9 voxels), with a search volume of 1373568 mm^3^ (21462 voxels, 198.7 resels; corresponding to 0.01 resels-per-voxel). In addition, as spatial smoothing can introduce a bias in the localization of reward-related brain activity (Sacchet & Knutson, 2014), we have calculated the mean resels-per-voxel specifically for the striatal area. This was done using a mask based on the activation patterns from the 'correct-versus-incorrect stops' contrast, created using the MarsBaR toolbox (Brett et al., 2002; <http://marsbar.sourceforge.net>), and produced a mean statistic of 0.015 resels-per-voxel.

**References**

Sacchet, M. D., & Knutson, B. (2013). Spatial smoothing systematically biases the localization of reward-related brain activity. NeuroImage, 66, 270-277. <https://doi.org/10.1016/j.neuroimage.2012.10.056>

**S.9 Trial sequence**

**Legend**: column 1; 5 = break, 0 = 0%, 1 = 22%, 3 = 33%; column 2: 0 = go, 1 = stop.

5 0

5 0

5 0

5 0

5 0

5 0

5 0

5 0

5 0

5 0

5 0

5 0

5 0

5 0

5 0

2 1

1 0

0 0

0 0

2 1

2 1

1 0

2 1

0 0

0 0

1 0

2 0

1 0

2 0

1 0

0 0

1 0

0 0

2 0

1 0

2 0

0 0

1 0

0 0

2 0

0 0

1 0

1 0

0 0

1 0

0 0

2 1

1 0

2 1

0 0

0 0

2 0

1 0

2 1

1 0

1 0

2 0

2 1

0 0

1 1

1 0

0 0

1 1

0 0

1 0

0 0

0 0

1 0

2 0

1 0

0 0

1 0

2 1

2 0

2 0

0 0

2 0

1 0

2 1

2 0

0 0

1 0

2 0

0 0

2 0

1 0

1 0

1 1

0 0

2 1

0 0

2 0

0 0

1 0

2 0

0 0

1 0

1 1

0 0

1 0

1 0

0 0

2 1

0 0

2 1

0 0

2 1

2 0

0 0

0 0

2 1

0 0

2 1

1 0

2 0

0 0

2 0

2 0

2 1

1 0

1 1

0 0

2 0

2 0

2 1

2 0

0 0

1 0

1 1

2 0

2 1

1 0

2 1

0 0

1 0

1 0

0 0

1 0

0 0

1 0

2 1

2 0

1 0

5 0

5 0

5 0

5 0

5 0

5 0

5 0

5 0

5 0

5 0

5 0

5 0

5 0

5 0

5 0

1 0

0 0

1 0

2 0

0 0

2 0

2 0

0 0

2 0

2 0

2 0

0 0

1 0

2 0

0 0

0 0

1 0

0 0

0 0

1 0

0 0

0 0

2 0

0 0

1 0

2 1

2 0

0 0

0 0

1 0

0 0

2 0

1 0

2 0

0 0

1 0

1 0

1 0

0 0

2 1

0 0

0 0

0 0

1 0

1 1

1 1

0 0

2 0

1 1

0 0

1 0

1 1

1 1

2 0

2 0

1 0

0 0

2 0

0 0

2 0

2 0

0 0

1 0

0 0

0 0

1 0

2 1

0 0

1 0

1 0

2 1

1 0

2 0

2 0

0 0

1 0

0 0

0 0

1 0

1 1

0 0

2 0

1 1

0 0

2 0

1 0

2 1

0 0

1 0

2 0

0 0

0 0

2 0

2 0

0 0

1 0

0 0

2 0

1 0

2 1

0 0

0 0

2 0

2 1

0 0

1 0

2 0

1 0

1 0

0 0

1 1

2 0

0 0

1 1

2 1

1 0

2 0

0 0

1 1

1 0

1 0

0 0

1 1

0 0

1 0

0 0

2 0

2 1

5 0

5 0

5 0

5 0

5 0

5 0

5 0

5 0

5 0

5 0

5 0

5 0

5 0

5 0

5 0
